# Statistical and thermodynamic analysis of the binding of trans-activation response–binding proteins to HIV-1 TAR RNA

**DOI:** 10.1016/j.jbc.2020.100067

**Published:** 2020-12-08

**Authors:** Jonghoon Kang, Albert M. Kang

**Affiliations:** 1 Department of Biology, Valdosta State University, Valdosta, Georgia, USA; 2 Princeton University, Princeton, New Jersey, USA

**Keywords:** TAR, trans-activation response, TBP, TAR-binding protein

In a recent article (1), the authors examine the binding of lab-evolved trans-activation response (TAR)–binding proteins (TBPs) to HIV-1 TAR RNA. Here, we show our analysis of the thermodynamic data of the binding that identifies three quantitative features of the binding, which may provide further insight into the interactions.

(1) The binding of TBPs to HIV-1 TAR RNA exhibits enthalpy–entropy compensation (2, 3), which suggests that the bindings of the six TBPs follow a common mechanism in the binding (Fig. 1*A*).

**Figure 1:**
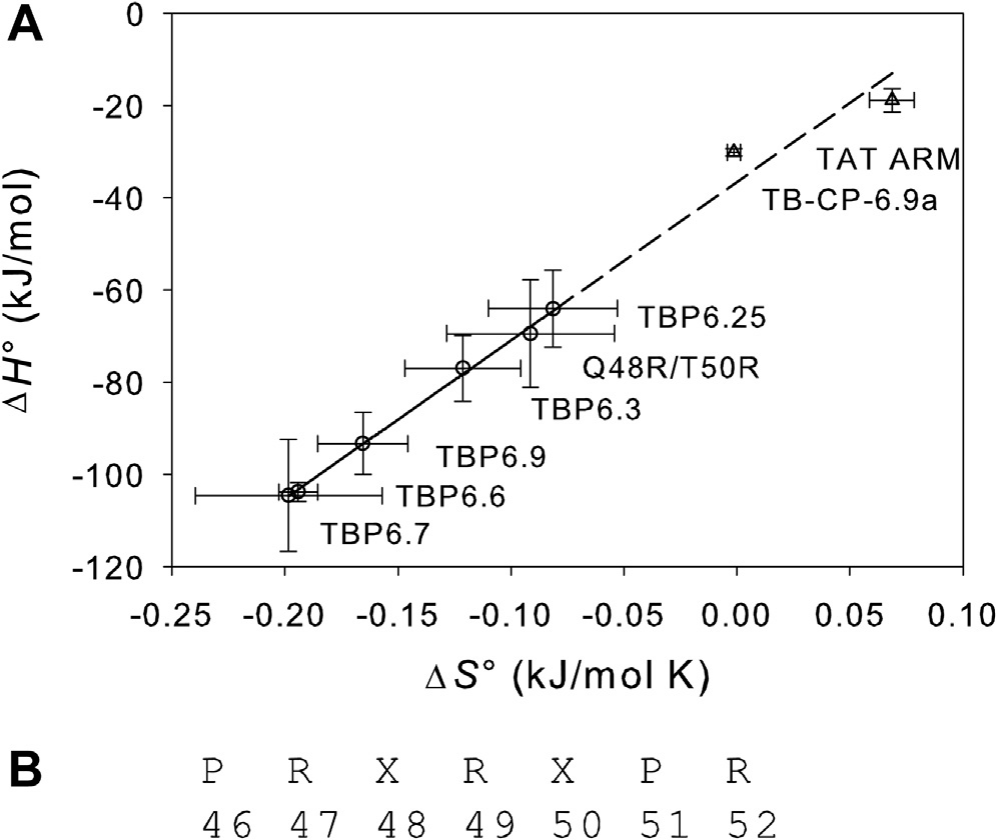
**Statistical and thermodynamic analysis of the binding of TBPs to HIV-1 TAR RNA.***A*, enthalpy–entropy compensation in the binding. The solid line is the best fit to the data without two outliers (Tat ARM and TB-CP-6.9a), Δ*H*° = 343.3 × Δ*S*° – 36.6 (*R*^2^ = 0.9972), and the dotted line is its extrapolation. *B*, the β2-β3 loop sequence of the lab-evolved TBPs. X indicates two positions of mutation, leading to 400 possible variations of the sequence. All statistical analyses were conducted using SigmaPlot (version 11; Systat Software). TAR, trans-activation response; TBP, TAR-binding protein.
(2) Two other variants of TBPs do not fit the linear regression (Fig. 1*A*), according to a regression diagnostic test using studentized deleted residuals (4). This suggests that the rest structure of TBPs other than the TAR-binding β2-β3 loop, which is absent in the two outliers, contributes to the binding.
(3) The Δ*G*° values of the six TBPs at 310.15 K calculated using Equation 1 are normally distributed, according to the Shapiro–Wilk normality test (W = 0.933, *p* = 0.607):

(1) ΔG°=ΔH°−TΔS°

where Δ*H*° and Δ*S*° are experimentally measured values reported in the original paper (1). Based on the statistical parameters, probability density of the Δ*G*° can be generated using Equation 2:

(2) $$p\left(\Delta G{}^{\circ}\right)=\frac{1}{\sigma \sqrt{2\pi }}\times \exp\;\left(-\frac{1}{2}\times {\left(\frac{\Delta G{}^{\circ}-\Delta G{{}^{\circ}}_{\text{mean}}}{\sigma }\right)}^{2}\right)$$

where *σ* = 1.9407 kJ/mol and Δ*G*°_mean_ = –41.3 kJ/mol. The distribution allows one to relate the probability to find a TBP variant to its binding affinity. For example, statistically expected maximum Δ*G*° of the highest-affinity variant of TBPs (Fig. 1*B*) is obtained by solving Equation 3 for *x* to be –46.8 kJ/mol:

(3) $$1-\overset{\infty }{\underset{x}{\int }}p\left(\Delta G{}^{\circ}\right)d\Delta G{}^{\circ}=\frac{1}{400}$$

## Conflict of interest

The authors declare that they have no conflicts of interest with the contents of this article.
